# Bilateral Rhegmatogenous Retinal Detachment After Uneventful Phacoemulsification Cataract Surgery: A Case Report and Literature Review

**DOI:** 10.7759/cureus.102644

**Published:** 2026-01-30

**Authors:** Shahmeer H Noori, Diya Baker, Syed Shahid, Doaa Kerwat, Nick Kopsachilis, Lorenzo Motta

**Affiliations:** 1 Medical Education, Hillingdon Hospital, Uxbridge, GBR; 2 Ophthalmology, East Kent Hospitals NHS Foundation Trust, Canterbury, GBR; 3 Ophthalmology, Homerton College, University of Cambridge, Cambridge, GBR; 4 Ophthalmology, Maidstone and Tunbridge Wells NHS Trust, Royal Tunbridge Wells, GBR; 5 Ophthalmology, University of Padova, Padua, ITA

**Keywords:** bilateral rhegmatogenous retinal detachment, cataract surgery, complication of cataract surgery, pseudophakia, rhegmatogenous retinal detachment (rrd)

## Abstract

A 72-year-old man developed a non-simultaneous bilateral rhegmatogenous retinal detachment (RRD), three months after bilateral sequential uneventful phacoemulsification cataract surgery performed through a 2.75 mm clear cornea incision by the same high-volume cataract surgeon. The patient was of Indian background and a high myope. Meticulous retinal evaluation had been performed preoperatively, and no retinal holes, tears, or other predisposing retinal abnormalities were discovered. Notably, posterior vitreous detachment (PVD) was absent bilaterally prior to cataract surgery but developed postoperatively preceding each RRD. The RRD was rectified by pars plana vitrectomy, retinopexy, and endocular tamponade.

## Introduction

Rhegmatogenous retinal detachment (RRD) refers to the detachment of the retina caused by a full-thickness retinal break, allowing liquefied vitreous to enter the subretinal space [1]. RRD is a well-known sight-threatening complication of cataract surgery, which represents the most performed elective surgical procedure in the developed world [1]. Although unilateral pseudophakic RRD is well described, bilateral RRD occurring within a short interval after cataract surgery is rare.

Across European population-based cohorts, pseudophakic RRD occurs approximately 2-4x more frequently than the comparative phakic population [2,3], with the incidence of cases rising alongside rising cataract surgery rates [4,5].

In the literature, such cases of pseudophakic RRD have been reported to occur as early as the first postoperative day [6], and as late as 23 years after surgery [7], with the latter occurring after clear lens exchange rather than cataract as the indication for lens removal. To date, we have identified one case of bilateral RRD following uncomplicated phacoemulsification, which occurred one month after immediate sequential bilateral cataract surgery (ISBCS), as described by Paquet et al. 2015 [8]. Our case is unique as the patient had sequential, uneventful phacoemulsification three weeks apart, then developed RRDs three months later, with a delay between eyes mirroring the three-week interval between surgeries. Furthermore, despite our patient being a male myope over 65 years of age, he did not have a posterior vitreous detachment (PVD), which is atypical.

## Case presentation

A 72-year-old Indian man was referred by his optometrist for cataract evaluation, reporting glare and night-driving difficulties. The binocular corrected distance visual acuity (CDVA) was 0.28 logarithm of the minimum angle of resolution (logMAR), with a CDVA of 0.38 logMAR in the right eye (RE) and 0.26 logMAR in the left eye (LE). The manifest spherical equivalents were -7.50 diopter (D) and -6.25 D, respectively. Familial and personal ocular history were unremarkable, and he was only on treatment with systemic antihypertensive medications.

Slit-lamp examination revealed moderate nuclear sclerosis with cortical changes bilaterally, more advanced in the right eye. The intraocular pressures were normal. Dilated fundus examination did not show pathologic abnormalities, with no evidence of complete posterior vitreous detachment (PVD). Given that same-day bilateral cataract surgery is not currently offered at our institution, he was scheduled for bilateral sequential phacoemulsification cataract surgery with a three-week interval. Optical biometry measured axial lengths of 26.94 mm and 27.39 mm, respectively.

**Figure 1:**
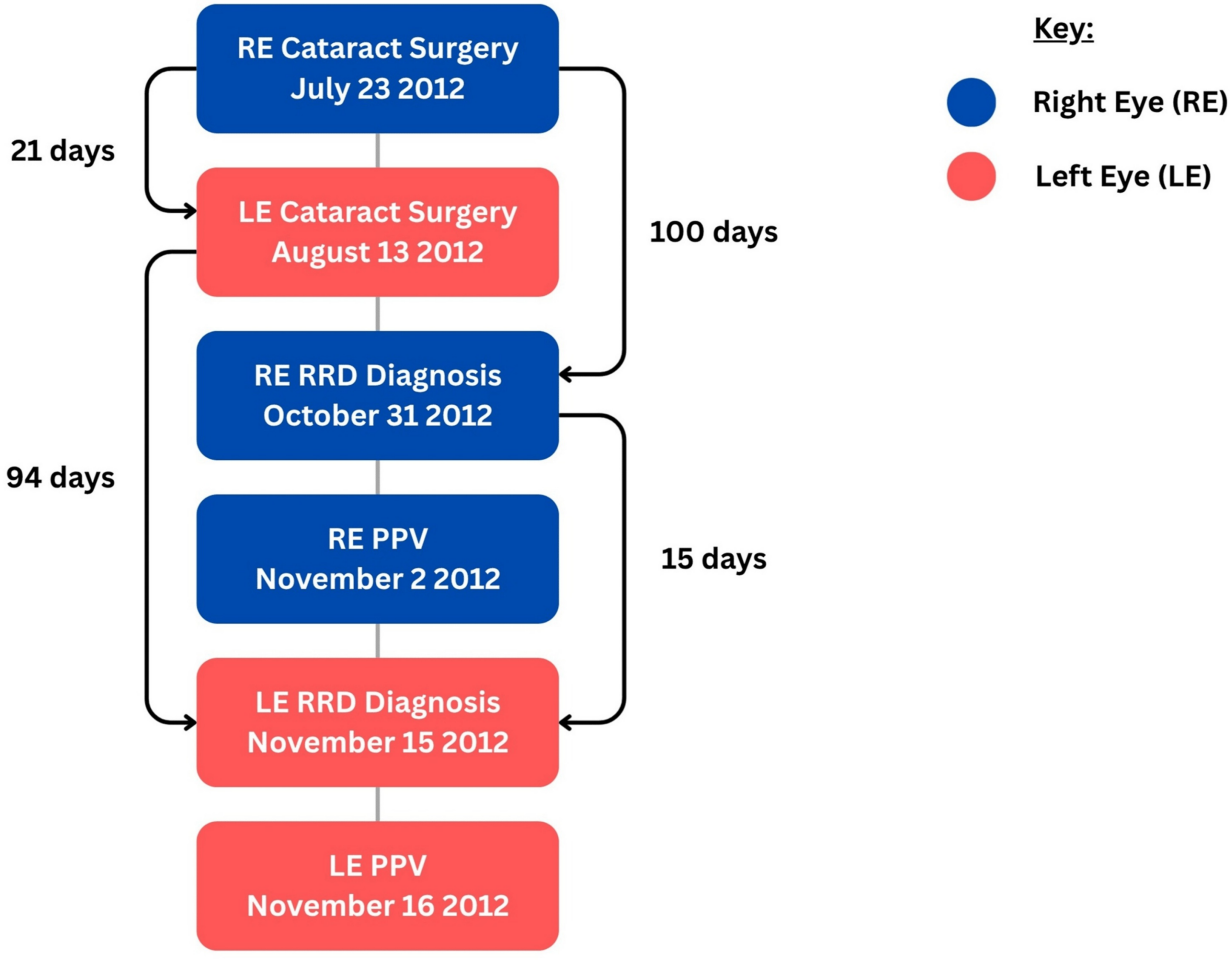
Timeline of surgical events and onset of bilateral rhegmatogenous retinal detachment. The right eye (RE, blue) underwent cataract surgery on July 23, 2012, followed by diagnosis of RE RRD 100 days later on October 31, 2012, and pars plana vitrectomy (PPV) on November 2, 2012. The left eye (LE, red) underwent cataract surgery 21 days after the RE on August 13, 2012, with LE RRD diagnosed 94 days later on November 15, 2012, and PPV the following day. The interval between RE and LE RRD onset was 15 days, closely matching the three-week interval between the initial cataract surgeries. This near-synchronous pattern highlights the temporal relationship between surgery and RRD development in this highly myopic patient without pre-operative posterior vitreous detachment. RRD: rhegmatogenous retinal detachment.

Both cataract surgeries were performed by the same experienced surgeon (GC) using a standard phacoemulsification technique: right eye on July 23, 2012, and left eye on August 13, 2012 (Figure 1). Both procedures were uneventful, each completed within 20 minutes. A single-piece acrylic hydrophobic intraocular lens (IOL) (Acrysof SN60WF; Alcon, Geneva, Switzerland) was implanted in the capsular bag, with intracameral cefuroxime for endophthalmitis prophylaxis. The refractive target was minimal myopia; IOL powers were +12.5 D and +10.5 D, respectively.

At the two-week postoperative examinations, the manifest refraction was plano in both eyes, and the unaided visual acuity improved to logMAR 0.06 (right) and logMAR 0.00 (left). A PVD was noted on examination of the right eye.

Given the acute PVD in a high-risk setting (high axial myopia, recent pseudophakia), a thorough peripheral retinal examination with scleral indentation was performed. No retinal tears, holes, or lattice degeneration were identified, alongside the absence of any vitreous haemorrhage and a negative Shafer’s sign. Given the detailed examination without any evidence of the aforementioned features, the patient was counselled regarding symptoms of retinal detachment and advised to return immediately if symptoms worsened, in keeping with the literature and current guidelines [9,10].

Just over three months afterwards (on October 31, 2012), the patient attended our emergency service reporting a five-day history of RE blurred vision and distortion, accompanied by photopsia. The CDVA had reduced to logMAR 0.60, and he was diagnosed with an inferior RRD associated with a shallow macular detachment after performing a detailed fundus examination with indentation. No retinal breaks were detected at this examination, nor at a further preoperative dilated fundoscopy performed by the vitreoretinal surgeon the next day.

Given the patient's high-risk profile: recent bilateral pseudophakia; high axial myopia; and RRD in the fellow eye, a thorough peripheral retinal examination with vitreous base indentation was performed in the left eye by the same vitreoretinal surgeon. No retinal tears, detachment, or predisposing lesions were identified, and no PVD was present.

The patient underwent right eye pars plana vitrectomy under sub-Tenon's anaesthesia two days later (November 2, 2012). Intraoperative examination identified a small superior peripheral break. Cryoretinopexy was applied, with internal drainage through an inferior retinotomy and 25% sulfur hexafluoride gas tamponade. The postoperative course was satisfactory at day one and day 11.

**Figure 2:**
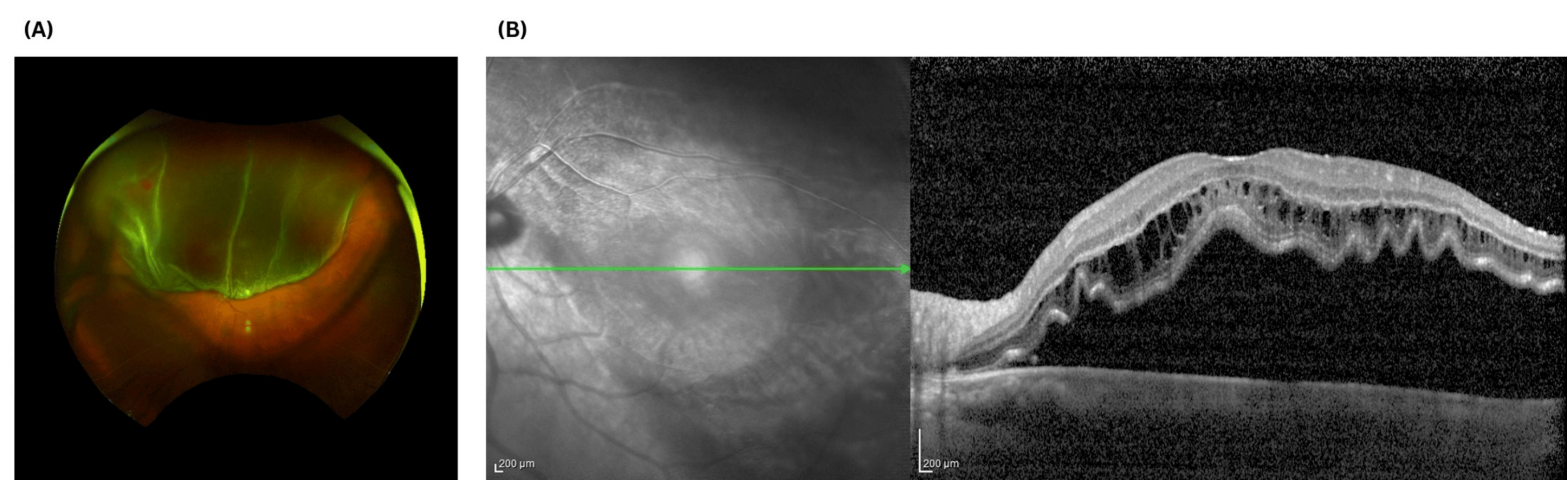
Multimodal imaging demonstrating a bullous macula-off rhegmatogenous retinal detachment (left eye). (A) Ultra-widefield fundus photograph of the left eye demonstrating a large superior bullous rhegmatogenous retinal detachment. The inferior retina remains attached, with a clear demarcation line between attached and detached retina. (B) Corresponding macular OCT showing elevation of the neurosensory retina with subretinal fluid beneath the fovea and corrugation of the outer retinal layers, confirming macular involvement. OCT: ocular coherence tomography.

On November 15, 2012, the patient attended again the emergency service with reduced vision in the LE (logMAR 1.10). Examination revealed a bullous macula-off RRD with an associated superotemporal retinal tear and an acute PVD (Figure 2). Given the established RRD with macular involvement, the patient underwent urgent pars plana vitrectomy the following day, using the same surgical technique as for the right eye.

At the most recent examination, CDVA was stable at logMAR 0.06 in the right eye and logMAR 0.82 in the left eye, reflecting the macula-on status of the right eye RRD versus the macula-off presentation in the left.

## Discussion

We present a case of bilateral sequential rhegmatogenous retinal detachment occurring within three months of uncomplicated phacoemulsification cataract surgery in a highly myopic male patient. While high axial myopia, male sex, and pseudophakia are each well-established independent risk factors for RRD [11,12], the clinical interest of this case lies not in the presence of these risk factors, which were clearly identified preoperatively, but rather in the striking temporal symmetry of bilateral detachment and the implications for fellow-eye surveillance in high-risk patients.

Cataract surgery is known to accelerate vitreous liquefaction and induce an earlier PVD, particularly in eyes without prior vitreous separation, contributing to RRD formation through surgically induced vitreoretinal traction [13]. The risk of pseudophakic RRD is two to four times higher than in phakic eyes [2,3], with male sex, younger age at surgery, and increased axial length being well-recognized risk factors [11,12]. Our patient exhibited two of these major risk factors: male sex and high axial myopia (27 mm), placing him at substantially elevated risk for pseudophakic RRD.

An interesting observation in this case was the absence of PVD at baseline despite the patient's age (72 years) and high myopia, a combination typically associated with early vitreous separation [14]. This suggests that the cataract surgery itself may have precipitated acute vitreous separation, consistent with the established pathophysiology of surgically induced PVD. In younger patients, stronger vitreoretinal adhesion increases RRD risk following cataract surgery [15]; our patient, despite his age, may have exhibited similar vitreous dynamics due to his pre-surgical attached vitreous state.

The divergent visual outcomes between eyes in this case illustrate the critical importance of macular status at presentation. The right eye, which presented with an inferior RRD and only shallow macular involvement, achieved a final CDVA of 0.06 logMAR. In contrast, the left eye presented with bullous macula-off RRD and marked neurosensory elevation on OCT, resulting in limited visual recovery (CDVA 0.82 logMAR). These findings are consistent with established evidence that the extent and duration of macular detachment are key determinants of visual prognosis following RRD repair [16].

The timing of RRD following cataract surgery follows a characteristic bimodal pattern: an early peak within the first year-driven largely by factors, such as myopia, younger age, and surgical complications, followed by a slower, linear increase over subsequent decades, reflecting progressive vitreous separation [11,17,18]. Our patient's detachments occurred at approximately 100 days (right eye) and 94 days (left eye) post-surgery, placing them within the early high-risk period. Notably, the three-week interval between the two RRDs precisely mirrored the three-week interval between the original cataract surgeries, suggesting a consistent lag time from surgical insult to retinal detachment in both eyes.

The most striking feature of this case is the temporal symmetry of bilateral involvement. Both eyes developed RRD approximately three months after their respective cataract surgeries, with the inter-eye interval for RRD onset (15 days) closely matching the inter-eye interval for surgery (21 days) (Figure 1). This synchronous pattern suggests that the pathophysiological processes leading to RRD were triggered simultaneously in both eyes at the time of their respective surgeries and progressed at comparable rates. To our knowledge, only one previous case of bilateral RRD following uncomplicated phacoemulsification has been reported, occurring one month after immediate sequential bilateral cataract surgery [8]. Our case extends this observation by demonstrating similar temporal relationships even with delayed sequential surgery.

The fellow eye in patients with unilateral RRD represents an important threat to vision. The Scottish Retinal Detachment Study found that 7.3% of RRD cases had bilateral involvement and 3-13% of unilateral RRD cases eventually involved the fellow eye, with fellow-eye detachments more common in pseudophakic individuals and those with higher myopia [19]. Our case illustrates that in high-risk patients (high myopia, pseudophakia), both eyes may follow similar pathophysiological trajectories following cataract surgery, underscoring the need for vigilant surveillance of the fellow eye.

The role of prophylactic laser retinopexy in the fellow eye following unilateral RRD remains an area of active investigation. While focal treatment of identified retinal breaks is well-established, prophylactic treatment of asymptomatic high-risk eyes lacks level I evidence [10,20]. However, emerging data suggest that prophylactic treatment may reduce the incidence of fellow-eye RRD, particularly in patients with lattice degeneration or high-risk retinal tears. In one study of giant retinal tears (GRT), a particularly high-risk subgroup, prophylactic 360° laser treatment reduced fellow-eye RRD from 43% to 13% [21]. Whether similar benefits apply to non-GRT pseudophakic eyes with high myopia remains unclear. In our case, a thorough peripheral retinal examination with scleral indentation was performed in the fellow eye at the time of the first eye's RRD diagnosis, and no treatable lesions were identified; however, RRD occurred 15 days later. This raises the question of whether more aggressive prophylactic strategies, such as encircling laser retinopexy, might be considered in high-risk bilateral pseudophakic patients with high axial myopia, though this remains to be validated in prospective studies.

This case reinforces several principles: (1) preoperative risk stratification with documentation of high axial myopia, male sex, and vitreous status during informed consent; (2) dilated fundus examination with scleral indentation as the reference standard for detecting peripheral retinal pathology; (3) thorough examination with appropriate safety-netting or follow up following acute PVD detection in high-risk patients; and (4) vigilant fellow-eye examination following unilateral RRD with consideration of prophylactic treatment when predisposing lesions are identified.

## Conclusions

In conclusion, this case of bilateral sequential RRD following uncomplicated phacoemulsification in a highly myopic male patient demonstrates the temporal relationship between cataract surgery and RRD development in high-risk individuals. The striking synchrony of detachment timing, with the inter-eye interval for RRD mirroring the interval between surgeries, suggests that the pathophysiological processes were initiated at the time of surgery in both eyes. This case underscores the importance of preoperative risk stratification, thorough informed consent, and vigilant postoperative surveillance in patients with high axial myopia undergoing cataract surgery. The marked difference in visual outcomes between the macula-on and macula-off presentations further emphasises the critical importance of early detection and treatment of RRD.

## References

[1] Ghazi NG, Green WR (2002). Pathology and pathogenesis of retinal detachment. Eye (Lond).

[2] Bjerrum SS, Mikkelsen KL, La Cour M (2013). Risk of pseudophakic retinal detachment in 202,226 patients using the fellow nonoperated eye as reference. Ophthalmology.

[3] Olsen T, Jeppesen P (2012). The incidence of retinal detachment after cataract surgery. Open Ophthalmol J.

[4] Madi HA, Keller J (2022). Increasing frequency of hospital admissions for retinal detachment and vitreo-retinal surgery in England 2000-2018. Eye.

[5] (2025). National Ophthalmology Database Audit: Seventh Annual Report of the National Cataract Audit. Ophthalmologists.

[6] Zimmerman R, Perlman JI (2002). Bilateral acute postoperative retinal detachment after cataract extraction: case report and review of the literature. J Cataract Refract Surg.

[7] Symeonidis C, Lamprogiannis L, Tsinopoulos I (2018). Bilateral identical intervals between phacoemulsification procedures performed 23 years before retinal detachment. Oman J Ophthalmol.

[8] Paquet P, Fischer MT, Distelmaier P, Mammen A, Meyer LM, Schönfeld CL (2015). Bilateral simultaneous retinal detachment in pseudophakia. Case Rep Ophthalmol.

[9] Richardson PS, Benson MT, Kirkby GR (1999). The posterior vitreous detachment clinic: do new retinal breaks develop in the six weeks following an isolated symptomatic posterior vitreous detachment?. Eye (Lond).

[10] Kim SJ, Bailey ST, Kovach JL, Lim JI, Vemulakonda GA, Ying GS, Flaxel CJ (2025). Posterior vitreous detachment, retinal breaks, and lattice degeneration preferred practice pattern®. Ophthalmology.

[11] Thylefors J, Jakobsson G, Zetterberg M, Sheikh R (2022). Retinal detachment after cataract surgery: a population-based study. Acta Ophthalmol.

[12] Morano MJ, Khan MA, Zhang Q (2023). Incidence and risk factors for retinal detachment and retinal tear after cataract surgery: IRIS® registry (Intelligent Research in Sight) analysis. Ophthalmol Sci.

[13] Ripandelli G, Coppé AM, Parisi V, Olzi D, Scassa C, Chiaravalloti A, Stirpe M (2007). Posterior vitreous detachment and retinal detachment after cataract surgery. Ophthalmology.

[14] Yonemoto J, Ideta H, Sasaki K, Tanaka S, Hirose A, Oka C (1994). The age of onset of posterior vitreous detachment. Graefes Arch Clin Exp Ophthalmol.

[15] Ivastinovic D, Schwab C, Borkenstein A, Lackner EM, Wedrich A, Velikay-Parel M (2012). Evolution of early changes at the vitreoretinal interface after cataract surgery determined by optical coherence tomography and ultrasonography. Am J Ophthalmol.

[16] Hazelwood JE, Mitry D, Singh J, Bennett HG, Khan AA, Goudie CR (2025). The Scottish Retinal Detachment Study: 10-year outcomes after retinal detachment repair. Eye (Lond).

[17] Day AC, Donachie PH, Sparrow JM, Johnston RL (2016). United Kingdom National Ophthalmology Database Study of cataract surgery: report 3: pseudophakic retinal detachment. Ophthalmology.

[18] Erie JC, Raecker MA, Baratz KH, Schleck CD, Burke JP, Robertson DM (2006). Risk of retinal detachment after cataract extraction, 1980-2004: a population-based study. Ophthalmology.

[19] Mitry D, Singh J, Yorston D (2012). The fellow eye in retinal detachment: findings from the Scottish Retinal Detachment Study. Br J Ophthalmol.

[20] Wilkinson CP (2014). Interventions for asymptomatic retinal breaks and lattice degeneration for preventing retinal detachment. Cochrane Database Syst Rev.

[21] Verhoekx JS, van Etten PG, Wubbels RJ, van Meurs JC, van Overdam KA (2020). Prophylactic laser treatment to decrease the incidence of retinal detachment in fellow eyes of idiopathic giant retinal tears. Retina.

